# Antiseptic Chitosan-Poly(hexamethylene) Biguanide Hydrogel for the Treatment of Infectious Wounds

**DOI:** 10.3390/jfb14100528

**Published:** 2023-10-19

**Authors:** Irine Rose Antony, Aathira Pradeep, Anoop Vasudevan Pillai, Riju Ramachandran Menon, Vasudevan Anil Kumar, Rangasamy Jayakumar

**Affiliations:** 1 Polymeric Biomaterials Lab, School of Nanosciences and Molecular Medicine, Amrita Vishwa Vidyapeetham, Kochi 682041, India; irineroseantony@gmail.com (I.R.A.); aathirapradeep29@gmail.com (A.P.); 2 Department of General Surgery, Amrita Institute of Medical Sciences and Research Centre, Amrita Vishwa Vidyapeetham, Kochi 682041, India; anoopvpillai@gmail.com (A.V.P.); rijurmenon@aims.amrita.edu (R.R.M.); 3 Department of Microbiology, Amrita Institute of Medical Sciences and Research Centre, Amrita Vishwa Vidyapeetham, Kochi 682041, India; vanilkumar@aims.amrita.edu

**Keywords:** chitosan hydrogel, poly(hexamethylene) biguanide, antibacterial activity, antiseptic, antibiofilm, infectious wounds

## Abstract

Topical wound infections create the ideal conditions for microbial colonization and growth in terms of moisture, temperature, and nutrients. When they are not protected, numerous types of bacteria from the internal microbiota and the external environment may colonize them, creating a polymicrobial population. Treatment of these wounds often necessitates the use of antibiotics that may have systemic harmful effects. Unlike antibiotics, topical antiseptics exhibit a wider range of activity and reduced systemic toxicity and resistance. In order to address this issue, we developed an antiseptic Chitosan-Poly (hexamethylene) Biguanide (CS-PHMB) hydrogel. The prepared hydrogel was characterized using Fourier Transform Infrared Spectroscopy (FTIR) and Scanning Electron Microscopy (SEM). SEM images showed the smooth morphology and characteristic FTIR peaks of PHMB and confirmed the incorporation of the antiseptic into the chitosan (CS) hydrogel. A Water Vapor Permeation Rate study confirms the moisture retention ability of the CS-PHMB hydrogel. Rheological studies proved the gel strength and temperature stability. The prepared hydrogel inhibited the growth of *S. aureus*, *P. aeruginosa*, *E. coli*, methicillin-resistant *Staphylococcus aureus* (MRSA), and *K. pneumoniae*, which confirms its antibacterial properties. It also inhibited biofilm formation for *S. aureus* and *E. coli*. CS-PHMB hydrogel is also found to be hemo- and cytocompatible in nature. Thus, the developed CS-PHMB hydrogel is a very potent candidate to be used for treating infectious topical wounds with low systemic toxicity.

## 1. Introduction

Wounds and ulcers are a fairly prevalent health issue worldwide. Skin deterioration creates the ideal conditions for microbial colonization and growth in terms of moisture, temperature, and nutrients [1]. When lesions are not protected, numerous species of bacteria colonize them to create a polymicrobial colony that can reach 100 million microbes per gram of skin in just 48 h [1,2]. A characteristic issue with wounds is infection. It is one of the leading causes of delay in wound healing, increasing the risk of patient morbidity and mortality [3]. Rising rates of acquired antibiotic resistance make infection management a more challenging task [4]. Consideration should be given to this global issue when preventing wound infections.

Research on antibiotic substitutes is critical since many populations still use antibiotics excessively [5]. Topically applied antiseptic treatments are often used to stop infections from developing in wounds; they work well against various fungi, yeasts, and bacteria and produce only modest levels of antimicrobial resistance [6]. Various antiseptic therapies are now employed in healthcare, and other delivery methods are being researched [3]. Many antiseptics for the treatment of infections have undesirable side effects or have inadequate delivery mechanisms despite extensive study in this area. Therefore, further investigation is needed on other antimicrobials and delivery mechanisms that might have fewer adverse effects. 

Poly (hexamethylene) biguanide (PHMB), commonly known as polyhexanide, is a broad-spectrum antiseptic [6,7], disinfectant [7], and preservative with demonstrated efficacy against a variety of bacteria. PHMB is a polymer with an average molecular mass of 2500 g/mol that is made up of a variety of PHMB chloride chains of various lengths [8]. With a pKa value of 10.96 [9], the PHMB biguanide group is positively charged at physiological pH levels and able to interact with anionic groups with ease [10]. It was originally believed to operate predominantly through the microbial membrane of the microorganisms [11]. It primarily functions by disrupting microbial membranes [12,13], where the interaction among cationic amine groups on the positively charged PHMB particle and anionic phospholipids on bacterial cell walls provided the basis for the antibacterial effectiveness [14]. However, new findings report that it selectively binds and condenses the DNA of bacteria, halting bacterial cell division. This antibacterial mechanism of action may help to explain why there is little chance of antimicrobial resistance to PHMB despite significant research since its development [7].

An optimal wound dressing maintains the area wet, liquefies antibacterial medications, and encourages the growth of fibroblasts. Since the hydrogels meet these requirements, they are a great option for applications at the wound [15,16,17]. Three-dimensional, cross-linked networks of water-soluble polymers make up hydrogels. Virtually any water-soluble polymer can be used to create hydrogels, which can have a diverse variety of chemical compositions and overall physical properties [18]. Drug-integrated hydrogels increase the distribution of pharmaceuticals at the target location in a prolonged release pattern, boosting the system’s overall effectiveness and decreasing the side effects brought on by some medications when supplied directly, retaining the therapeutic index [19].

Chitosan is one good material for hydrogel preparation out of many (bio)polymers. Chitosan displays positive charges in physiological settings because it contains quaternary ammonium salt groups [20]. It is also simple to physically and chemically modify to create immunological activation, the promotion of wound healing, and antibacterial activities due to many active functional groups on the molecular chain [21,22]. Many wound-healing processes have been facilitated and managed by the inclusion of medicines in chitosan hydrogel compositions. Contrary to other therapies, the use of these hydrogels can stop the loss of bodily fluid, maintain the moisture of the wound surface, hasten healing, and serve as a barrier against bacterial infections [23,24].

CS incorporated with various antiseptic agents has been reported to treat wound infections. Bandages made of CS and octenidine dihydrochloride (Ocd) are one such wound dressing with promising antibacterial and antibiofilm activity [25]. A possible low-dose topical antibacterial PVA-based dressing with increased antimicrobial action has been reported using an antiseptic agent, PHMB [26].

**Figure 1:**
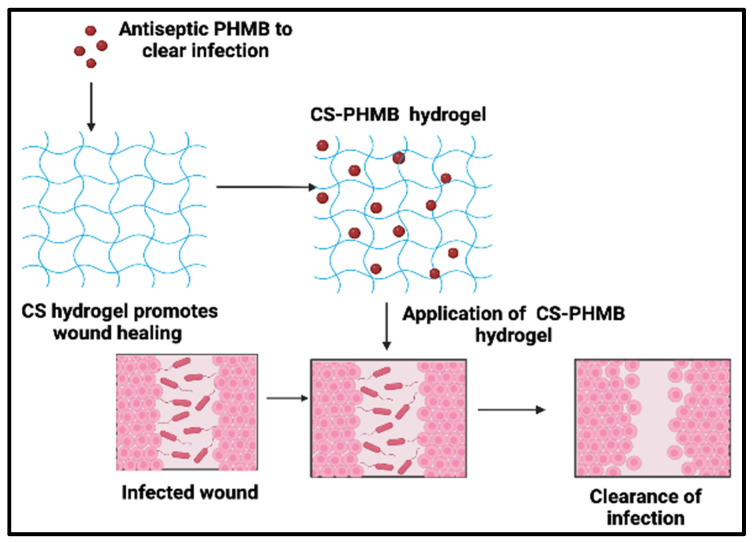
Schematic representation for strategy of wound treatment using CS-PHMB hydrogel.

In light of the previous considerations, we created and investigated a CS-PHMB hydrogel for the treatment of infectious wounds (Figure 1). This could reduce the problems associated with infections at the location of the wound and decrease the detrimental effect of microbes on the healing process.

## 2. Materials and methods

### 2.1. Materials

Chitosan (DDA-89.4%) was bought from the Japanese company Koyo Chemicals Co., Ltd., Japan. Fischer Scientific, USA provided the NaOH and acetic acid. PHMB was acquired from Simson Pharma Limited, Mumbai, India. The Microbiology Laboratory at Amrita Hospital in Kochi, India, provided the ATCC isolates of bacterial strains like *Escherichia coli* (ATCC 25922), *Klebsiella pneumoniae* (ATCC 700603), *Pseudomonas aeruginosa* (ATCC 9027), *Methicillin-Resistant Staphylococcus aureus* (MRSA) (ATCC 43300) and *Staphylococcus aureus* (ATCC 25923). LB Agar and LB Broth were acquired from HiMedia, India. Gibco provided Dulbecco’s modified Eagle medium, Fetal Bovine Serum, Trypsin, Amphotericin B, and Tryphan Blue, which were obtained from Lonza. Sigma provided the resazurin powder. We bought calcium chloride, sodium chloride, and sodium hydrogen phosphate from Merck, USA.

### 2.2. Synthesis of CS and CS-PHMB Hydrogel

**Figure 2:**
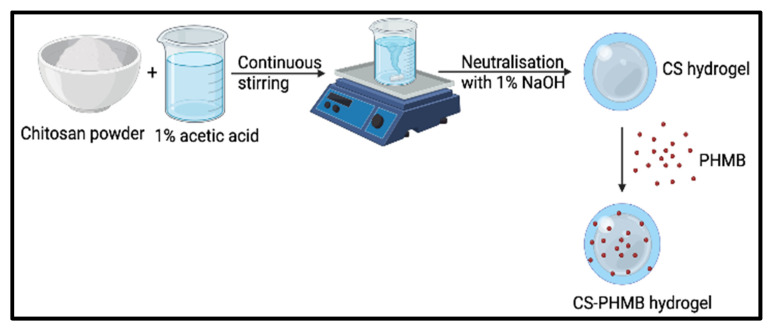
Schematic illustration for the synthesis of CS-PHMB hydrogel.

To make 1.5 (*w*/*v*%) CS hydrogel, 1.5 g of CS was suspended in 100 mL of a 1% acetic acid solution. The mixture was stirred continuously at 8000 rpm until it was fully dissolved. After that, the prepared solution was gradually neutralized with 1% NaOH till its pH reached a range of 7–7.4. In order to obtain the pure gel, the hydrogel is repeatedly washed with distilled water to eliminate the extra NaOH and drained overnight. A total of 500 μL of 2% PHMB stock solution was diluted with 500 μL milli-Q water to obtain 1% PHMB solution. Mixing 100 μL of PHMB solution with 1 g of previously prepared CS hydrogel yielded the final CS-PHMB hydrogel (Figure 2).

### 2.3. Characterisation Studies

The developed CS and CS-PHMB hydrogel samples were dried in air and sputter coated with gold before being submitted to scanning electron microscopy on a JEOL JSM-6490LA, Japan to examine the morphology at a 1000× magnification and a 15 kV acceleration voltage. To further characterize the CS and CS-PHMB hydrogel, FTIR analysis (Shimadzu IR Affinity-1S, Japan) was carried out. A mixture of 165 mg of KBr and 2 mg of lyophilized gel systems were used to create pellets, and the spectra were recorded between 4000 and 500 cm^−1^.

#### Injectability and Inversion Test of CS and CS-PHMB Hydrogel

In order to determine the injectability of the created hydrogels, they were dispensed onto a 1 mL syringe, and their type of flow was physically evaluated. Inversion tests were also carried out for 0 to 72 h by inverting undisturbed vials containing 2 g hydrogel.

### 2.4. Rheological Studies of CS and CS-PHMB Hydrogel

All rheological measurements were performed using a Malvern Kinexus pro rheometer (Malvern instruments, UK) with parallel plates having a 20 mm diameter and a 0.5 mm gap between lower and upper plates. All tests were conducted at 37 °C utilizing a Peltier heating device and an ambient enclosure to simulate the physiological temperature. Frequency sweep analysis, amplitude sweep analysis, flow curve analysis, and temperature stability were evaluated for the gel systems to define the nature, strength, and stability of developed hydrogels.

To start, an amplitude sweep was performed at 25 °C with a constant frequency and then varying shear rate to identify the gel strength and liquid/solid dominant behavior and their Linear Viscoelastic Region (LVER). The frequency sweep between 0.1 to 10 Hz in the LVER region determined the elastic (G′), viscous (G′′), and phase angles (δ) of the gels. The flow curve analysis was performed to test the shear thinning properties of the prepared gels at various shear speeds (γ), ranging from 10^−1^ s to 10^2^ s. The gel stability from room temperature to body temperature was further tested using a thermal stability test by altering temperature from 25 to 50 °C. The goal of this investigation was to comprehend the gel’s stability and ability to move from ambient temperature to the physiological temperature of a human.

### 2.5. Water Vapor Permeability Test

The measurement of water vapor permeability rate (WVPR) was conducted using a modified version of the ASTM International standard method ASTM E96-93 [27]. To perform the test, hydrogel films were placed onto the surface of a 50 mL beaker and filled with 25 mL of distilled water. The glass beaker, along with the hydrogel on top, was initially weighed (*Wi*) and then placed inside a desiccator containing silica gel. After being kept in the desiccator for 24 h, the vial was re-weighed (*Wf*). The WVPR value (expressed in units of grams per square meter per day, g m^−2^ day^−1^) was computed using the following formula:

$$\mathrm{WVPR}=\frac{Wi-Wf}{A*T}$$

where A is the area of the opening beaker and T is the time period of the experiment.

### 2.6. In Vitro Hemolysis Assay of CS and CS-PHMB Hydrogel

Red blood cells (RBCs) were obtained by centrifuging the human blood at 500× *g* for 5 min while it was in a vacutainer tube containing 3.2% sodium citrate. Then, 1 mL of the diluted RBC suspension, 100 mg of CS, and CS-PHMB hydrogel were placed in appropriate tubes and then incubated at 37 °C for 3 h before being centrifuged at 500 rcf for 5 min. Positive control consisted of 1 mL of diluted RBC suspension mixed with 100 μL Triton-X, and 1 mL of diluted RBC suspension was mixed with 100 μL saline as a negative control. The optical density of the supernatant after centrifugation was assessed at 540 nm. The proportion of hemolysis was calculated using the formula below:

$$Hemolysis\;\%=\frac{OD\;sample-OD\;negative}{OD\;positive-OD\;negative}$$

### 2.7. In Vitro Cytocompatibility of CS and CS-PHMB Hydrogel

Alamar blue assay was employed to estimate the cytocompatibility of CS and CS-PHMB hydrogel for fibroblast cells. In a 48 well plate, L929 cells were seeded (20,000 cells/well), and then 20, 40, 60, 80, and 100 mg of produced gels were added. Cells were cultured in DMEM media with 10% FBS, 1% antibiotic, and antimycotic. Alamar blue reagent was used to check the viability at 24 h of incubation at 37 °C and 5% CO_2_. By using a plate reader (Biotek Power Wave XS, USA), the OD values were read at 570 nm and 600 nm to determine the extent of the color shift in the Alamar Blue solution after 4 h of incubation. The positive control was cells treated with Triton X-100 at a concentration of 0.1% *v/v*.

### 2.8. In Vitro Drug Release Profile of CS and CS-PHMB Hydrogel

The drug release was monitored for 7 days by incubating 100 mg of CS hydrogel with 1.5% PHMB in 1X PBS (1 mL) at 37 °C. The dissolution media was collected and replaced by fresh PBS at predetermined time points. PHMB release was determined using UV spectrophotometer at 236 nm [28]. The percentage of PHMB release was calculated using the formula

$$\mathrm{PHMB}\;\mathrm{released}\;\%=\frac{\mathrm{Amount}\;\mathrm{of}\;\mathrm{PHMB}\;\mathrm{released}\;\mathrm{at}\;\mathrm{specific}\;\mathrm{time}\;\mathrm{point}}{\mathrm{Total}\;\mathrm{amount}\;\mathrm{of}\;\mathrm{PHMB}\;\mathrm{entrapped}\;\mathrm{within}\;\mathrm{the}\;\mathrm{hydrogel}}\times 100\;$$

### 2.9. In Vitro Antibacterial Activity of CS and CS-PHMB Hydrogel

The antibacterial action of CS and CS-PHMB hydrogel was tested against *K. pneumoniae*, *E. coli*, *P. aeruginosa*, MRSA, and *S. aureus*. On the LB agar plate, 100 μL of each strain’s overnight bacterial cultures were spread out. The wells of the plate were filled with 100 mg of CS and CS-PHMB hydrogels. Then, the plates were stored for incubation for 24 h at 37 °C in an erect position.

Additionally, the antibacterial action was evaluated for the clinical isolates of MRSA, *E. coli*, and *K. pneumoniae* as well. Bacterial strains were streaked horizontally on agar plate, and a vertical strip of Whatmann filter paper was laid through the streaks. The filter paper was covered with the hydrogels, and plates were then incubated at 37 °C. By measuring zone of inhibition, the microbial activity produced on the ATCC and clinical strains was evaluated.

### 2.10. Antibiofilm Studies of CS-PHMB Hydrogel

LB broth with 0.5% glucose was used to make separate cultures of *E. coli* and *S. aureus*, which were then added to the wells of a 48 well plate along with 2% glucose and 1 mL of fresh LB broth. Sterilized coverslips were kept slanted in the wells and left there for 48 h of incubation. After that, fresh LB broth having 2% glucose was used to substitute the media, and corresponding wells were then added with prepared CS and CS-PHMB hydrogel, followed by a 24 h incubation. The media was discarded from the wells after 24 h, and the coverslip washed thrice with saline before being overnight dried at 37 °C. The dried coverslips were rinsed in saline following staining for 5 min with 0.1% acridine orange. The coverslips were photographed using a fluorescence microscope after being washed and dried. Using ImageJ (version 1.53t, NIH, USA) software and a 3D plugin, the photos were transformed into 3D surface plots. Another set of tests comprised placing the coverslips that had been stained with acridine orange in a well plate and using 70% acetone to extract the acridine orange-bound biofilm. Using a microplate reader, the absorbance of the eluted fractions was measured at 495 nm.

### 2.11. Statistical Analysis

The values were calculated and expressed as mean ± standard deviation (n = 3). To determine the statistical significance, one-way ANOVA was utilized. *p* values below 0.05 are considered to be significant and were indicated by the symbol ****.

## 3. Results and Discussion

### 3.1. Preparation and Characterization of CS and CS-PHMB Hydrogel

The chitosan hydrogel was made using the neutralization method. When exposed to alkali like NaOH, the charge density and protonated amine groups in the chitosan solution will help in neutralization [29,30]. Faster gelation is achieved by using lower concentrations of chitosan and larger concentrations of NaOH. NaOH molecules are said to be carried by the chitosan hydrogel as they diffuse through it [31]. At room temperature, the prepared hydrogels exhibited good stability. The concentration of PHMB to be added into the hydrogel was determined by its antibacterial action based on Minimum Inhibitory Concentration against the bacteria tested. A total of 1% PHMB showed good antibacterial activity against all bacteria. Concentration less than 1% showed no antibacterial activity against *P. aeruginosa*. Thus, CS-PHMB hydrogel was made by evenly mixing 1% of PHMB into the CS hydrogel.

**Figure 3:**
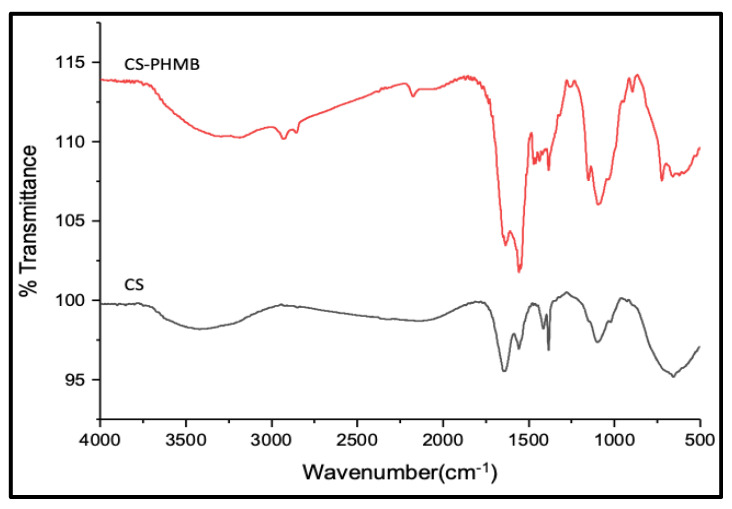
FT-IR spectrum of CS and CS-PHMB hydrogel.

FTIR spectra of CS (Figure 3) show a characteristic absorption band at 2900–2880 cm^−1^ due to -C-H stretching. CS also shows an absorption band at 1642 cm^−1^, which corresponds to the N-acetyl glucosamine group [32]. The main characteristic absorption band of PHMB occurs at 2000–2400 cm^−1^, and the spectra obtained from the final CS-PHMB hydrogel showed an absorption band at 2369 cm^−1^ corresponding to the nitrogen-related vibrations of PHMB [32]. Moreover, the absorption bands at 2853 and 2924 cm^−1^ correspond to symmetric and asymmetric -CH stretching, whereas the absorption bands at 1627 and 1547 cm^−1^ correspond to the -NH bending of imine groups of PHMB [33]. Thus, the FTIR spectra confirm the presence of both CS and PHMB absorption bands in CS-PHMB hydrogel. 

**Figure 4:**
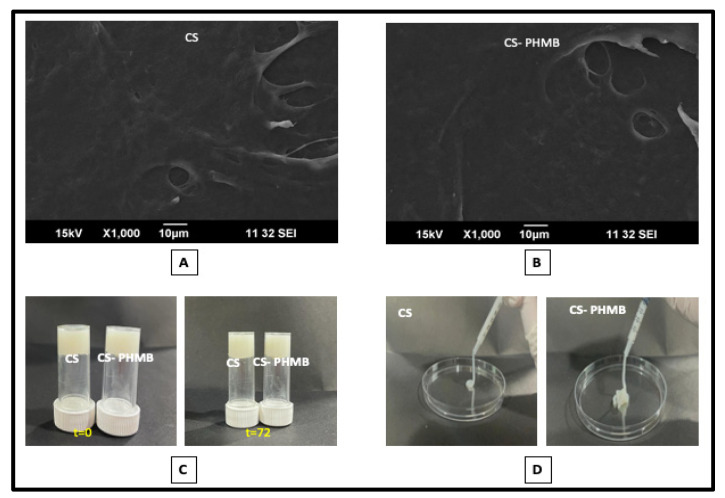
(**A**,**B**) SEM images, (**C**) injectability, and (**D**) inversion test studies of CS and CS-PHMB hydrogel.

SEM analysis of the CS and CS-PHMB hydrogels had a smooth surface morphology, as shown in Figure 4A,B. The addition of PHMB in CS hydrogel did not alter the surface morphology of the CS gel. The gel was unable to flow when subjected to gravity, even after 72 h, as seen by the inversion test (Figure 4C), which may relate to the inability of the prepared hydrogel to flow without an external shear, allowing it to stay on the infection site without slipping. While performing injectability studies (Figure 4D), the prepared gel systems maintained their natural characteristics and flowed smoothly without breaking, which may be related to the shear-thinning properties of the hydrogel. This helps in easing the application of the gel precisely at the wound site. This finding confirms the fabrication of a smooth and injectable hydrogel that can be easily injected into infected wounds.

### 3.2. Rheological Studies of S and CS-PHMB Hydrogel

**Figure 5:**
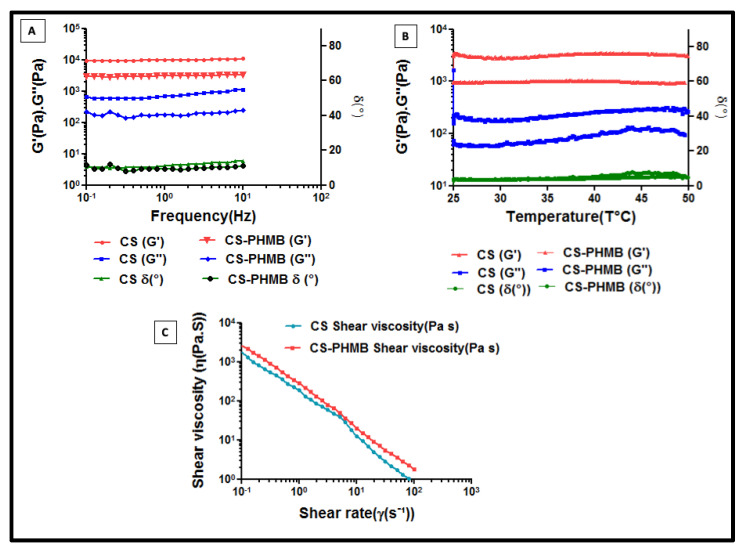
Rheological properties of CS and PHMB-CS gel systems. (**A**) Frequency sweep, (**B**) Temperature sweep, and (**C**) Flow curve analysis.

The LVER of the gel was recorded using the amplitude sweep. Materials can elastically deform within the LVER zone and, if the deformation is eliminated, revert to its initial state [34]. An analysis of the gel’s behavior over time was performed using a frequency sweep in the LVER region (Figure 5A). The gel was exposed to a frequency range of 10^−1^ to 10^1^ Hz in order to determine its G’. The elastic modulus (G’) of both hydrogels was found to be higher than their viscous modulus (G″). This indicates that they are predominantly solid dominant in nature. Any material should have a predominately solid nature alongside a liquid component in order to have a gel-like character. The phase angle remained constant over the analyzed frequency range, confirming the gel-like nature of the material. The gel has a phase angle (δ) less than 20°, indicating that the gel can elastically deform like a solid. For pure solids, δ = 0° and for liquids δ = 90° [35]. The gel was stable in the temperature range of 25–50 °C during temperature stability analysis (Figure 5B). During the frequency and temperature sweep, G’ values were stable over the analyzed range. This suggested that the stability of CS-PHMB gel would remain relatively constant even after being injected into the wound site [35]. The flow curve test (Figure 5C) was used to analyze the shear-thinning properties of the gel. The viscosity η was tested at various shear speeds (γ), ranging from 10^−1^ s to 10^2^ s. The curve showed that as the shear rate increased, the viscosity decreased owing to the shear thinning behavior of the hydrogels. Thus, the CS-PHMB hydrogel shows a solid dominant nature with a liquid component to exhibit a gel-like nature [35].

### 3.3. Water Vapor Permeability Test

Skin is a semi-permeable membrane in control of regulating water and oxygen transport as well as cellular vitality. For a wound to heal properly, transdermal water loss must be prevented [36]. In order to prevent the wounded region from drying out, the dressing should have the appropriate WVPR. The WVPR of inherently hydrophilic CS was found to be 1296.493 ± 26.854 g/m^2^/day. Withthe addition of PHMB in the prepared CS hydrogel, the WVPR of the final CS-PHMB hydrogel increased to 1455.125 ± 30.255 g/m^2^/day. Commercial wound dressing products have WVPR in the range of 90–2893 g/m^2^/day [37]. The higher WVPR of the CS-PHMB hydrogel could be due to the incorporation of hydrophilic PHMB in the biopolymer. This will create an indirect path through which the water molecules can enter to maintain optimum water content at the wound site [38,39] and promote wound healing.

### 3.4. In Vitro Hemolysis Assay of CS and CS-PHMB Hydrogel

**Figure 6:**
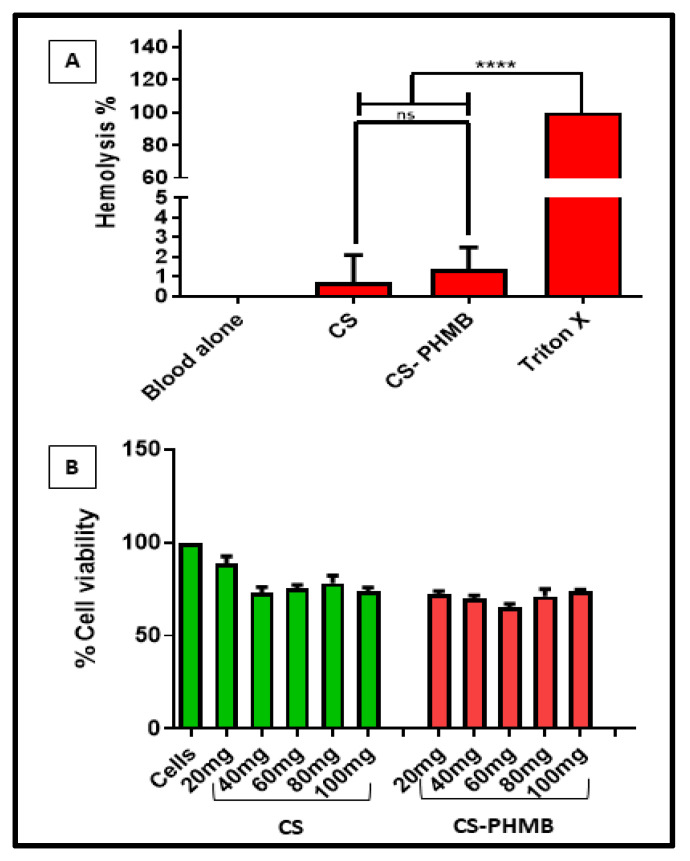
In vitro (**A**) hemocompatibility of CS and CS-PHMB hydrogel and (**B**) cytocompatibility assay of CS and CS-PHMB hydrogel. The *p* value < 0.0001 is indicated by **** and ns denotes non-significance.

An essential component of biological safety for all medicinal formulations is blood compatibility. After incubation for 3 h along with RBCs, the proportion of hemolysis was found to be 0.678% and 1.400% for CS and CS-PHMB hydrogels, respectively, in comparison to triton-X and saline (Figure 6A). This shows that the prepared CS-PHMB hydrogels are hemocompatible as their hemolysis rate is very low, i.e., lower than 5%, which is the accepted threshold for biomaterials interacting with blood [40].

### 3.5. In Vitro Cytocompatibility of CS and CS-PHMB Hydrogel

The cell viability studies were performed by keeping different amounts of the samples with L929 cells for 24 h, and then their % viability was calculated. It was noted that after 24 h of incubation, the % viability of 20, 40, 60, 80, and 100 mg of the CS gel were found to be 88.665 ± 9.976, 73.047 ± 7.324, 75.224 ± 5.200, 78.174 ± 10.2006 and 73.788 ± 5.200% respectively. The viability of CS-PHMB hydrogel was reduced to 72.278 ± 4.023, 70.009 ± 3.874, 65.298 ± 4.359, 71.189 ± 9.501 and 73.527 ± 2.799%. It has been reported that PHMB by itself shows cytotoxicity on dermal fibroblasts, keratinocytes, and osteoblasts [41]. However, the incorporation of PHMB in inherently biocompatible CS does not cause a significant reduction in cell viability of the CS-PHMB hydrogel (as shown in Figure 6B) in comparison to the CS alone hydrogel.

### 3.6. In Vitro Drug Release Profile of CS and CS-PHMB Hydrogel

**Figure 7:**
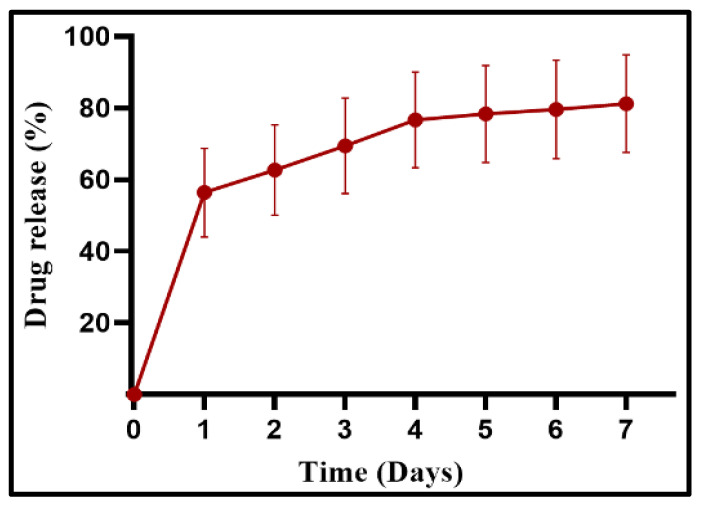
Drug release profile of PHMB from CS-PHMB hydrogel.

The release profile of PHMB from CS-PHMB hydrogel shows a prolonged release pattern, as shown in Figure 7. The initial burst release on day one was followed by a gradual and sustained release, resulting in an 81.23% release of PHMB on the seventh day. The burst release can be attributed to the weak bonding between CS and PHMB. The release is then slowed down due to the deterioration of weaker physical forces (van der Waals’ forces and hydrogen bonding) in the CS-PHMB hydrogel system, as well as surpassing the surrounding diffusion barriers [28]. Another group has reported the initial burst release of PHMB within the first 12 h, followed by a sustained release up to 20 days in porous silk fibroin sponges loaded with PHMB [42].

### 3.7. In Vitro Antibacterial Activity of CS and CS-PHMB Hydrogel

**Figure 8:**
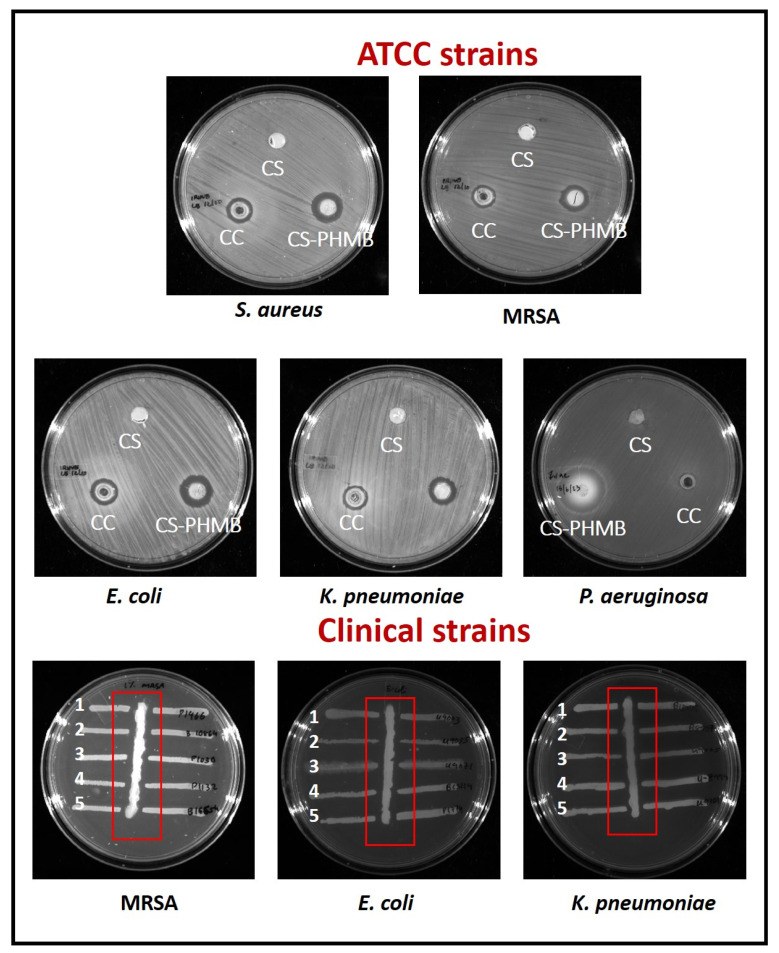
Antibacterial activity of CS and CS-PHMB hydrogel against *S. aureus*, *E. coli*, *K. pneumoniae*, *P. aeruginosa*, and clinical isolates of MRSA, *E. coli* and *K. pneumoniae.* 1–5 indicates the different isolates.

By using the agar well diffusion method, the antibacterial action of the produced hydrogels (Figure 8) was examined for ATCC strains of Gram-positive bacteria like *S. aureus* and MRSA and Gram-negative bacteria like *E. coli*, *K. pneumoniae*, *P. aeruginosa*, and clinical strains of MRSA, *E. coli* and *K. pneumoniae*.

After a 24 h incubation period, CS-PHMB hydrogel showed a distinct zone of inhibition against the ATCC strains. The same outcome was seen when testing clinical strains, where CS-PHMB hydrogel inhibited bacterial growth and CS hydrogel showed no zone. CS-PHMB hydrogel showed a better zone of inhibition compared to the commercial control (Maxicocel). These outcomes might be due to the concentration of PHMB around the proteins of the phospholipid bilayer to alter the bacterial cell membrane environment, followed by membrane damage, allowing the internal constituents of the cell to flow out [43]. Smaller ions like K^+^ and Na^+^ flow out first and kill the bacterium [44]. PHMB may utilize a cationic penetrating peptides mechanism to penetrate the phospholipid bilayer [45]. It was also reported that phospholipid binding causes PHMB translocation across the bilayer, resulting in DNA interactions with the polymer to block DNA replication [4]. Local application of this CS-PHMB hydrogel in the area of injury can inhibit bacterial development without creating any systemic damage.

### 3.8. In Vitro Antibiofilm Assay of CS-PHMB Hydrogel

**Figure 9:**
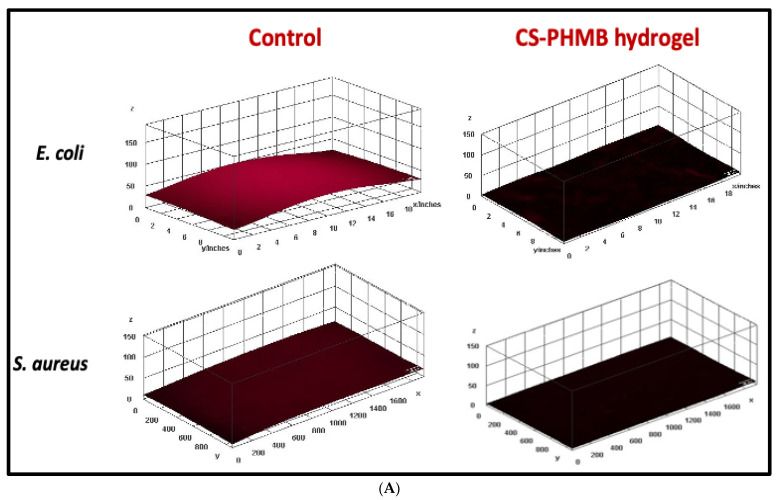

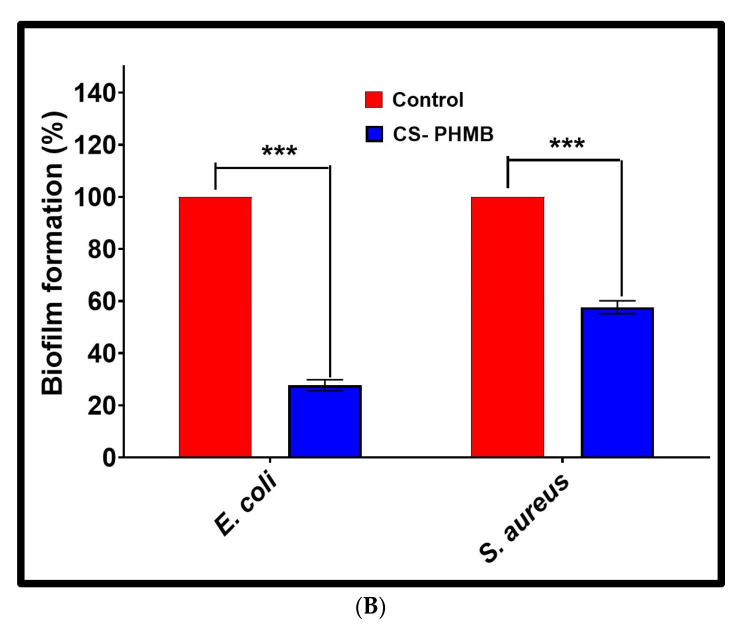
(**A**) 3D images of biofilm inhibition by CS and CS-PHMB hydrogel. (**B**) Quantification data of biofilm inhibition by CS and CS-PHMB hydrogel. *p* values less than 0.001 are denoted by ***.

The bacterial biofilm may have a significant part in the slow healing of chronic wounds. Biofilm inhibition of CS-PHMB hydrogel was studied by acridine orange staining, which binds to the nucleic acid of the bacteria on the biofilm-grown coverslips. Under a fluorescent microscope, the biofilm growing on the coverslips shows bright red fluorescence and very little fluorescence in the absence of biofilm growth. The 3D surface plots of fluorescent microscopic images from the biofilm inhibition assay are depicted in Figure 9A. The existence of biofilm is shown by higher red-colored fluorescence in the CS control group, while the suppression of the biofilm is indicated by reduced to nearly nil fluorescence in the CS-PHMB treated group. Quantification of the study (Figure 9B) indicates significant inhibition of biofilm formation for both *E. coli* and *S. aureus.* For *E. coli*, biofilm formation was reduced to 27.71 ± 2.11%, whereas, in the case of *S. aureus*, it was reduced to 57.61 ± 2.52% when compared to that of the control.

Biofilms are made of Extracellular polymeric substances (EPS), including polysaccharides, extracellular DNA, proteins, and lipids. Biofilms also contain extracellular DNA, whose function is to encourage bacterial adherence to surfaces and to maintain the structural integrity of the EPS structure [45,46]. Despite being a large molecule, PHMB also has the ability to bind with DNA electrostatically [47]. Thus, interactions between PHMB and the EPS structure may have contributed to the antibiofilm effects.

## 4. Conclusions

In this work, we have synthesized and characterized CS-PHMB hydrogel for the treatment of infectious topical wounds. The prepared hydrogels show a smooth morphology and are injectable in nature. From the rheological studies, it was found that the elastic modulus of the hydrogel was higher than the viscous modulus and stable over the analyzed ranges. Flow curve analysis confirms the stability and shear thinning properties of the CS-PHMB hydrogel and is stable at physiologic temperature. WVPR test of the CS-PHMB hydrogel shows its ability to retain moisture at the wound site to assist wound healing. The prepared CS-PHMB hydrogel was also hemo- and cytocompatible. CS-PHMB hydrogel showed burst release of the drug and potent antibacterial activity against *E. coli*, *P. aeruginosa*, *K. pneumoniae*, *S. aureus*, and MRSA and clinical strains of *E. coli* and *S. aureus.* Therefore, this CS-PHMB hydrogel can be used topically for the clearance of both Gram-positive and Gram-negative wound infections. The biofilm formation % dropped significantly for clinical strains of *E. coli* and *S. aureus*, respectively, when using CS-PHMB hydrogel. All these data suggested that prepared CS-PHMB hydrogel is a very potent candidate to be used for treating infectious wounds. Further in vivo investigations need to be performed in the future prior to introducing the CS-PHMB hydrogel in a clinical setting.

## Data Availability

The data are available upon a reasonable request to the corresponding authors.
